# Nonhuman and Nonhuman-Human Communication: Some Issues and Questions

**DOI:** 10.3389/fpsyg.2021.647841

**Published:** 2021-09-23

**Authors:** Irene M. Pepperberg

**Affiliations:** Department of Psychology, Harvard University, Cambridge, MA, United States

**Keywords:** interspecies communication, symbolic reference, nonhuman communication, animal cognition, Grey parrot cognition

## Abstract

Deciphering nonhuman communication – particularly nonhuman vocal communication – has been a longstanding human quest. We are, for example, fascinated by the songs of birds and whales, the grunts of apes, the barks of dogs, and the croaks of frogs; we wonder about their potential meaning and their relationship to human language. Do these utterances express little more than emotional states, or do they convey actual bits and bytes of concrete information? Humans’ numerous attempts to decipher nonhuman systems have, however, progressed slowly. We still wonder why only a small number of species are capable of vocal learning, a trait that, because it allows for innovation and adaptation, would seem to be a prerequisite for most language-like abilities. Humans have also attempted to teach nonhumans elements of our system, using both vocal and nonvocal systems. The rationale for such training is that the extent of success in instilling symbolic reference provides some evidence for, at the very least, the cognitive underpinnings of parallels between human and nonhuman communication systems. However, separating acquisition of reference from simple object-label association is not a simple matter, as reference begins with such associations, and the point at which true reference emerges is not always obvious. I begin by discussing these points and questions, predominantly from the viewpoint of someone studying avian abilities. I end by examining the question posed by Premack: do nonhumans that have achieved some level of symbolic reference then process information differently from those that have not? I suggest the answer is likely “yes,” giving examples from my research on Grey parrots (*Psittacus erithacus*).

## Introduction

The songs of whales and birds, the roars of lions and bellows of elephants, the pant-hoots and grunts of apes, the squeaks of mice and croaks of frogs – humans have long been fascinated by the meanings of nonhuman communications systems. And those examples involve only the auditory mode – what about the flashes of lightning bugs or the scent systems that make dogs stop and sniff every few feet on their daily walk? Lest any doubt exist about the longstanding and widespread nature of such interest amongst even nonscientists, one need only cite examples such as the legend of King Solomon’s ring, which purportedly enabled him to communicate at will with all the birds and beasts in his realm (Lorenz, 1952); the historic lore of Native Americans, who supposedly could change into various animals and thus share their lives (Rasmussen, 1972), or the many children’s books on the subject (e.g., the Dr. Doolittle series; Lofting, 1920). Darwin (1871) in particular wrote at length about the similarities between human and nonhuman communication systems but provided scant guidance for deciphering the latter. Realistically, in his era little experimental research was possible that could have discovered potential meaning in nonhuman systems or their relationship to human language: no one yet had the appropriate tools to determine whether nonhuman signals expressed anything more than emotional states or conveyed actual bits and bytes of concrete information.

Such research began in earnest in the first half of the 1900s but was extremely limited in scope, as the methodology was hardly more advanced than in Darwin’s day. Nice (1943) and Saunders (1951), for example, were among the first to quantify and contextualize birdsong in a scientific manner and demonstrate the inherent complexity in various species’ systems. They employed musical notation and detailed field notes to describe the vocalizations of birds and various contexts in which such vocalizations were used in species such as song sparrows. Researchers, such as Marler (1956) and Thorpe (1958), continued this work with a variety of species, and pioneered use of the Sonagraph^©^, which gave plots of time vs. frequency (kHz) that enabled these songs to be analyzed in a myriad of ways. However, these researchers were a small minority among scientists who were, particularly in the early half of the 20th century, more interested in collecting skins and categorizing species than in studying behavior – especially communication.

Not until the latter half of that century would tools and techniques be designed that allowed humans to engage in the detailed analyses and formal experiments that would provide the first real insights into the realm of animal communication systems, primarily with respect to birdsong. High-quality microphones and tape recorders (“portable” only in the sense that they could be transported into the field!) allowed researchers to acquire recordings of actual songs as they were sung in nature; the Sonagraph^©^ and these recorders also enabled researchers to choose and play specific songs back to the birds to see what effects hearing these had on behavior. And, thus began the study of nonhuman communication systems in earnest…

## Birdsong, Primate Calls, and Vocal Learning

The second half of the 20th century was an especially exciting time for the study of nonhuman behavior. In 1973, for the first time ever, three ethologists (Nikolaas Tinbergen, Konrad Lorenz, and Karl von Frisch) won the Nobel Prize in Physiology or Medicine, a solid recognition of the validity of the field. Notably, the work of each of them involved, to greater or lesser degrees, nonhuman communication. From the standpoint of someone like myself, interested in vocal interactions (particularly in birds), the wealth of new information that was beginning to accrue was almost overwhelming. Although earlier researchers (e.g., Howard, 1920) had proposed that song had specific purposes – territorial defense and mate attraction – they lacked clear evidence to support these claims. Now researchers were able to acquire data about the actual meaning and function of avian signals (Pepperberg, 2020c): Dilger (1956) showed that thrushes used song to distinguish subspecies from one another as well as to defend their territories from competitors. Marler (1952) discovered local dialects in the songs of chaffinches, which steered Thorpe (1958) to the study of their song acquisition and led both scientists to the investigation of vocal learning – if song was innately specified, all birds of the same species should sound almost exactly the same; thus, the existence of dialects suggested that differences were acquired. (I will discuss song learning in detail later.). Weeden and Falls (1959) revealed that ovenbirds used their songs for individual recognition; Konishi (1964), for example, found that the trill part of the song served that purpose in Oregon juncos. Many researchers (e.g., Krebs et al., 1978; Yasukawa, 1981) used playbacks to determine how individual recognition was used to discriminate neighboring males (those birds who had pretty much defined their abutting territories and who used countersinging to keep the status quo) from stranger males (those who were not local and posed a serious disruptive threat to the status quo). Marler (1960) provided some of the earliest evidence that songs were also used for mate attraction and was supported by somewhat later studies such as those by Kroodsma (1976) and Krebs (1977) on multiple uses of song, and how different songs could be used for different purposes (e.g., see Catchpole, 1983; later Kroodsma et al., 1989). Marler (1961) and Smith (1963) separately systematized the analyses of nonhuman communication systems, drawing heavily on avian studies. Studies in some non-songbirds – parrots – showed that these birds also exhibited individual vocal recognition and alarm calling, suggestive of sentinel behavior, that alerted the flock to various flying predators (Lawson and Lanning, 1980; Levinson, 1980).

Note that many of the same techniques were being used to study vocal behavior in nonhuman primates (e.g., Struhsaker, 1967; Seyfarth et al., 1980a,b), which led to the claim that vervet monkeys had referential, vocal signaling – different calls for different types of predators (i.e., their argument was that each signal denoted – referred to – a specific entity; I will discuss the meaning of “reference” and various levels of referential behavior more fully in a separate section). The vervet study stirred up considerable controversy as to the extent or level of the referentiality exhibited (Pepperberg, 2020c): Nay-sayers argued that, unlike humans, vervets could not “discuss” predators outside of alarm calling in their presence (see Bickerton, 1990), and even Marler (1974) noted that the calls could simply be context-dependent (i.e., merely indicating “danger above” vs. “danger below” instead of referring to specific predators). Note that the controversy continues (see Fischer, 2011; Wheeler and Fischer, 2012; Townsend and Manser, 2013), even Seyfarth and Cheney eventually backed off somewhat in the strength of their claims (see Price et al., 2014). Whatever the level of reference, however, the calls of both birds and primates did contain *content* – warning signals. Referentiality, as I will discuss later, is an especially fraught topic.

Despite advances in the realm of nonhuman primate communication, the study of birdsong retained a very special status in the research community for one interesting reason: Marler (1970a) and Thorpe (1974), based on pioneering studies by researchers like Nottebohm (1966, 1970), had found that many avian species – like humans and *unlike* nonhuman primates – were vocal learners and, like humans, have lateralized brain areas responsible for such acquisition. These researchers therefore suggested that birds could be better models than nonhuman primates for studying the evolution of language, despite being more evolutionarily distant from humans than nonhuman primates. Specifically, vocal learning allows for innovation and adaptation, characteristics that release communication from rigid, innately specified responses to stimuli. Although no one then (or even now) could claim that birds’ songs have the kind of syntactical and semantic complexity of human language, researchers did demonstrate numerous parallels between song and language (a brief summary follows; for full reviews see, for example, Doupe and Kuhl, 1999; Peters and Nowicki, 2017; also, see Beecher, in this collection of papers).

Starting with the earliest stages of acquisition, both birds (Marler, 1970a) and humans (e.g., Oller et al., 1976) engage in a period in which they “babble” – that is, they experiment with the sounds that will ultimately become the building blocks of their repertoire. Moreover, in both cases, the babbling itself consists of stages, such that progress toward the adult behavior can be monitored (e.g., Marler, 1970b; Oller, 1978; de Boysson-Bardies et al., 1989). Of particular interest is that both birds and humans initially produce an extremely wide variety of sounds – formally termed *overproduction* – of which only a subset will eventually be used in their communication systems. This initial overproduction enables the possibility, respectively, of producing many songs/dialects and all human languages. Over the course of maturation, a winnowing occurs, based on cultural input, to focus learning on the most relevant sounds in their communicative environment (e.g., Rice and Thompson, 1968; Doupe and Kuhl, 1999).

Another common aspect involves the issue of what were initially called “critical periods” for acquisition – limited, tightly time-specified windows during which exposure to the adult system was considered necessary if learning was to occur (e.g., Lenneberg, 1967; Marler, 1970a). Such windows are now considered to be much less time-constrained, given that subsequent research has demonstrated how, for example, exposure to intense, live, social interactions rather than auditory tapes can greatly extend the period for acquisition (e.g., Fromkin et al., 1974; Baptista and Petrinovich, 1984, 1986; Grimshaw et al., 1998; Beecher and Brenowitz, 2005). Thus, the preferred phrase to describe these intervals is now “sensitive phases.” Nevertheless, for both humans and birds, early exposure allows the most *facile* development. Of note, however, are those avian species such as canaries, starlings, and parrots that are considered “open-ended learners” (Adret-Hausberger et al., 1990; Nottebohm, 2006); these birds have the capacity to acquire new vocalizations throughout their lives. A related aspect involves the issue of bilingualism – in humans, the acquisition of a second language; in birds, the acquisition of a second dialect or even the song of another species (allospecific song). Early studies on birds, again using only auditory tapes (Marler, 1970a), argued that allospecific song acquisition was prevented by an innate template, and also argued that the same template likely prevented acquisition of multiple dialects; early studies on humans argued that bilingualism was limited by the same critical period that was thought to constrain first language (Johnson and Newport, 1989). Again, subsequent studies demonstrated that, depending upon the type and extent of input, later acquisition can indeed occur – that the length of the sensitive phase can be extended, quite significantly, in both birds and humans (e.g., Baptista and Petrinovich, 1984, 1986; Hartshorne et al., 2018).

The use of birds as models for language evolution was made even stronger by the studies on the neurobiology and neuroanatomy of the vocal learning system in birds and humans. For a detailed but still concise review, see Jarvis (2019). Briefly, although the capacity for vocal learning most likely arose independently in birds (parrots, songbirds, and hummingbirds) and mammals (which include bats, elephants, and marine mammals as well as humans), specific analogous brain areas and connections that are responsible for that ability have been found in humans and avian vocal learners (note Colquitt et al., 2021) and particularly in vocal open-ended learners; these areas and connections are lacking in closely-related species that are not vocal learners. According to Jarvis (2019, p. 4):

“the common ancestor of vertebrates had a brainstem pathway for production of innate vocalizations with limited vocal plasticity… In some species, the forebrain motor learning pathway then duplicated and formed a vocal motor learning pathway with weak direct projections to the brainstem vocal motor neurons. Thereafter, this forebrain vocal motor learning pathway expanded in neuron numbers causing greater density of neurons in the forebrain, moved outside of the motor learning pathway, and gained dense direct projections to brainstem vocal motor neurons. Finally, the vocal learning pathway then duplicated one or more times and took on additional specialized gene regulation and connections, resulting in the advanced vocal learning pathways we find in parrots and in humans.”

Although none of this detailed neurobiological information was known in the 1970s, enough about the avian brain had been learned (e.g., Nottebohm, 1970) to serve as the basis for the extensive research on avian vocalizations that led to these discoveries. The main points, of course, are that (a) for three avian lineages, there exist hundreds (parrots and hummingbirds) to thousands (oscines) of species in which vocalizations are learned, (b) learning is possible because of specific neuroanatomical areas and their functions, and (c) for those and many more species, vocalizations do contain information that (based on the actions of the receiver) appears to be processed in meaningful ways, even if humans have so far been unable to decipher much beyond those related to territorial defense, threats, mating and, in later studies, how the use of different songs or singing patterns in different contexts relates to degrees of aggressive behavior (see, for example, Smith, 1996; Beecher et al., 2000). I will henceforth use the term “meaningful” to define the vocalizations described in (c) above. I am not arguing that the behavior of the receiver is simply (possibly in a stimulus–response manner) triggered by the signal or manipulated by the signaler, but rather that the receiver *processes* (i.e., actively decodes and then integrates into prior and current knowledge) the information in the signal (plus possible other relevant input) and then decides on an appropriate course of action (note Seyfarth et al., 2010 for a discussion of this point).

## Avian-Human Differences

Despite all these exciting parallels, some striking differences were found to exist between avian and human communication systems. Bird vocalizations demonstrate levels of *functional* reference (i.e., alarm calls, songs for mate attraction vs. territorial defense all encode *relatively* specific information about eliciting events, but must be processed with respect to the full context in which they are emitted); however, they apparently lack the kind of semanticity and syntax found in human speech. Specifically, Marler (1977) has characterized birdsong as having only a “phonological syntax”; that is, as a system in which the acoustic elements are arranged according to set rules in order to attract mates, deter rivals, and defend territory. One can argue that such is also true of combinations of physical and vocal displays used to extend the meaning of songs in a repertoire, especially when a bird has only a single song (e.g., Smith, 1996). However, the content of any specific *element* (for example, a note or syllable) does not (at least to our current knowledge) appear meaningful (i.e., in the sense of a human word; see below for additional information). Furthermore, such ordering is not a requisite for all avian species: For some vocal learners, note and syllable order is crucial for meaning and function; for other species it is not (see review in Weisman and Ratcliffe, 1987). Note that a more appropriate characterization of the latter might be that of *phonetic patterning* (Collier et al., 2014). However, for species in the former group (e.g., chestnut-sided warblers, *Dendroica pensylvanica*, Byers, 1995), particular songs – which consist of the same, but re-ordered, acoustic elements – *do* have different functions. Such distinctions may also be the case for certain bird calls – in particular instances, when the order of the elements is altered, birds fail to respond in playback tests (Suzuki et al., 2019). These data suggest that some sorts of rules for the production and comprehension of vocalizations may exist for some avian species, even though they are separated from humans by over 300 million years of evolution (Hedges et al., 1996). Human language, however, depends on the semantic meaning of each element of each sentence, as well as its hierarchical structure (e.g., Berwick et al., 2012). Moreover, elements of birdsong or calls are not (again, as far as humans have been able to determine at present!) routinely combined to form *novel* meanings for *novel* situations, as are human utterances. The only evidence for such avian combinations involves single behavioral instances and not specific individual vocal elements; for example, a Florida scrub jay (*Aphelocoma coerulescens*) once combined alarm calls associated with, respectively, hawks and snakes in the presence of a perched owl (Hailman and Elowson, 1984).

Thus, despite all these parallels between human language and birdsong, and arguments about nonhuman primate calls, researchers still were unable to determine the *extent* to which nonhuman communication was meaningful. To reiterate, experiments had shown that nonhuman signals *did* convey a certain amount of content – upon hearing certain signals, receivers knew what species was vocalizing, whether they should take evasive action from a predator and often what kind of action, whether another individual was trying to take over their territory or simply maintain a mutual boundary, whether a male was still searching for a mate – but this content involved basic behavioral states and concrete information. What seemed to be lacking was the type of *abstract* meaning that characterized human signals; for example, the ability to describe or comment upon something that was not physically present or is imaginary or the ability to combine signals in *novel* ways to describe *novel* situations.

## Interspecies Communication

It was in the latter half of the 1960s through the 1970s – somewhat congruent with the period described above – that parallel sets of experiments involving both human language and nonhumans were begun. Here, researchers’ goals were not to crack the code that nonhumans were using among themselves, but instead were to try to teach a variety of species, through multiple modalities, to communicate directly with humans, with the idea that such interspecies communication would be “a possible window on the minds of animals” (Griffin, 1976, ch. 7). The intent was to demonstrate that, given such training, the various species would develop true symbolic reference and at least some level of rule-governed performance (a basic form of syntax); the underlying premise was that such behavior could not be instilled *de novo*, but that it could be taught only if it were based on some already existent abilities (or even predispositions). Specifically, the extent of success in instilling symbolic reference would provide some evidence for, at the very least, some cognitive underpinnings of referential nonhuman communication systems: even if humans could not find ways to unequivocally demonstrate aspects of human language in nonhumans’ native communication systems, absence of evidence might not necessarily prove evidence of absence, and with training, maybe latent abilities could be brought to light. Thus, by using interspecies communication, humans would begin to explore the cognitive and linguistic capacities of nonhumans.

Not surprisingly, such studies began with the great apes – creatures with a close phylogenetic relationship to humans (reviewed in Marks, 2005), and cetaceans – creatures with large brains relative to their body sizes (reviewed in Ridgeway, 1990). First came several failed attempts to train nonhumans to speak English (e.g., Lilly, 1967; Kellogg, 1968; Hayes and Nissen, 1971). Later, more successful experiments followed: The ape studies involved chimpanzees (*Pan troglodytes*) trained with American Sign Language (ASL; Gardner and Gardner, 1969), magnetized plastic chips (Premack, 1971), and a computer-based system (Rumbaugh, 1977). A gorilla (*Gorilla gorilla*, Patterson, 1978) and an orangutan (*Pongo pygmaeus*, Miles, 1978) were also trained with ASL and Signed English. The early dolphin (*Tursiops truncatus*) studies involved vocal whistle imitation and responses to arm signals (Herman, 1980).

Intrigued by all these studies and armed with knowledge of the birdsong research plus the clear mimetic ability of parrots, I decided, in 1977, to determine whether a Grey parrot could also engage in a related form of interspecies communication (Pepperberg, 1999) – one using the sounds of English speech. The odds were not in my favor: Parrots, as noted above, are separated from primates by over 300 million years of evolution (Hedges et al., 1996); they were thought to be mindless mimics (Lenneberg, 1967); at the time were presumed to lack anything like a cortical area (the earliest confirmation of such a functionally homologous area was not published until Reiner et al., 2004; Jarvis et al., 2005, although some glimmerings did already exist: see Cobb, 1960; Portman and Stinglin, 1961; Nauta and Karten, 1971); and previous studies using the standard conditioning techniques of the time had failed to instill communicative competence in a variety of mimetic avian species (e.g., Mowrer, 1950, 1952, 1954; Grosslight and Zaynor, 1967). A subsequent study (Todt, 1975), however, recognized the importance of social interaction in training techniques (specifically, use of a modeling procedure called the model/rival or M/R technique, in which two humans demonstrated the types of interactive vocal behavior that the subject is to learn) and eschewed conditioning. Todt (1975) demonstrated some level of success in that his Grey parrot subject engaged in a limited number of dialogues with its human trainer. Such findings suggested that the psychologists’ previous failures to achieve meaningful communication with their birds (or to find any level of complex cognitive processing) might be a consequence of inappropriate training techniques, rather than any inherent lack of ability in their psittacine subjects, and that vocal human-parrot communication might be possible. Specifically, I argued that by using additional, fairly new information on social learning (e.g., Bandura, 1971; Todt, 1975) and what little was known about parrot communication at the time (e.g., Power, 1966a,b; Nottebohm, 1970; Busnel and Mebes, 1975; Wickler, 1976; for a detailed review, see Pepperberg, 1999), I could adapt this M/R technique and achieve some level of referential acquisition.

Interestingly, all attempts at interspecies communication using human-based systems succeeded to some extent. Results from the different laboratories were divergent, but complementary (Pepperberg, 2017). The studies using variants of sign language (Gardner and Gardner, 1969; Miles, 1978; Patterson, 1978) allowed the apes to exhibit flexibility and innovation; because their system was also used with humans, it allowed direct comparisons of communicative acquisition between child and ape. Alternatively, the use of an original no-fault training procedure that rewarded associations of plastic chips with physical objects and enabled sophisticated tests of both trained and untrained chimpanzees (Premack, 1971) provided less information about communication skills than the ASL-based studies but began to elucidate how acquisition of symbolic reference could affect cognitive processing. A computer-controlled system, using a chimpanzee-sized version of a Skinner box and an artificial “language” (Rumbaugh, 1977), provided information about which basic concepts could be acquired *via* associative learning and how such learning could still allow for innovation. Herman (1980) began to show that dolphins could respond to specific cues with specific actions that demonstrated referential comprehension. My parrot started to use the sounds of English speech to identify objects, materials, colors, and shapes (Pepperberg, 1981). We believed that we were gaining valuable insights into the origins of referential communication: if creatures separated by so many years of evolution and with remarkably different-looking brains could all acquire some level of symbolic reference and regular ordering of those symbols, would not that imply the existence of some common origin or convergence?

Our resulting publications triggered approbation and condemnation in equal amounts, including from each other (Pepperberg, 2017). Unlike most controversies in science, however, discussions that began in scholarly journals (e.g., Bronowski and Bellugi, 1970; Lachman and Mister-Lachman, 1974; Terrace et al., 1979) were soon abandoned. Arguments and counter-arguments were prominently portrayed in the media, culminating in a chaotic meeting at the New York Academy of Science in the Spring of 1980 (Marx, 1980; Wade, 1980; Sebeok and Rosenthal, 1981). Important issues got lost in the resulting brouhaha, specifically questions as to, for example (Pepperberg, 2017, p. 182):

“…what were the actual hallmarks of language, what might the apes’, dolphins’ and parrot’s abilities tell us about language evolution and cognitive processing, what stages did children go through en route to full language, how did codes such as ASL differ from spoken language and were these differences important? (Note that at one point some scientists questioned if ASL was even a real human language; a full analysis hadn’t been published until Stokoe, 1978).”

The result was that most (although not all) of us lost our funding and abandoned our emphasis on the extent to which nonhumans could acquire the elements of human language; we focused instead on using whatever levels of interspecies communication that we had instilled in our subjects to examine various forms of cognitive processing that could be specifically examined *via* symbolic reference – a single feature of human language. Thus, despite our abandonment of studies of how much and how many aspects of human-based language a nonhuman could acquire, the issue of *reference* remained basic to our findings.

## Referential Communication

Deacon (1997, p. 44), in his influential book *The Symbolic Species*, argues that symbolic reference is “the central riddle in the problem of language origins” and claims that, except for those few nonhumans trained in interspecies communication, it is what separates human and nonhuman minds. Whether or not one agrees with his overall thesis, his assertions with respect to the importance of reference cannot easily be ignored. His point is that “reference” is not present when a label (or a sign) is simply *associated* with something (e.g., as is a red button that, when hit, delivers food whereas a green one does not) but is present if the label actually stands for something in a unique manner that is independent of context (e.g., “blue” describes the color of a pansy, a berry, a certain wavelength of light, as well as the skin color of a well-known but *fictional* entity). Once an individual understands symbolic reference at this level, the information content of symbols can be manipulated independently of their physical instantiation. Thus, finding the extent to which nonhumans’ communication systems – whether natural or acquired – involve reference can be central to (i.e., affect) how they process information, and thus to their cognitive abilities (Premack, 1983). This point is one that I will discuss at length below; for now, the discussion concerns how to determine reference (i.e., and levels thereof) in a nonhuman system.

Separating reference from association is not a trivial task. Initially, biologists and linguists formulated “design features” of human language (e.g., Hockett and Altmann, 1968), in which issues, such as *arbitrariness*, *interchangeability*, *displacement*, and *semanticity*, play a role in designating what constitutes signals that are referential – rather than simple associations. Arbitrariness eliminates signals that cannot be separated from the referent, such as the meowing of a cat to designate a cat, unlike “c-a-t” in English, or “c-h-a-t” in French – if you do not know the language, you do not know the meaning. Interchangeability eliminated signals that travel only from sender to receiver, or vice-versa, like a pheromone that signals sexual receptivity; one sex emits it, the other attends, and that is the extent of its use. Displacement eliminates signals that are used only to indicate something that is physically present, that is, signals that cannot be used to describe something in the past or future, or that one would wish to be present; a food grunt does not describe the antelope that was consumed yesterday. Semanticity eliminates signals that do not designate something specific; in a Piercian sense (see Pierce, 1978), smoke indicates the presence of fire, and can be seen to “represent” fire, but can also imply many things related to fire, such as a type of meat being grilled, and thus “smoke” is not considered to be the label for fire. As noted above, researchers initially claimed reference for vervet alarm calls (Seyfarth et al., 1980a,b); after examining the issues of reference, however, these calls seem to be more indexical, in that they indicate the immediate presence of something and the type of response that one must take. It would seem, however, that such level of use is completely sufficient for the vervets; the issue for humans who are trying to establish exact levels of reference (see below) is the difficulty of, for example, designing experiments to determine whether nonhuman communication involves material such as telling one another to avoid the drinking hole near the anthill today because somebody saw a leopard there this morning. The few attempted experiments to examine possibilities of that nature (e.g., Cheney and Seyfarth, 1985) have multiple alternative explanations for the resulting data: should a vervet produce an alarm call at the sight of a gazelle carcass that humans have deceptively cached in a leopard-like manner? Although the presence of the dead animal may mean that a leopard is nearby and thus that a call is appropriate, the carcass also likely means that the leopard would already have plenty of food and will not be hunting a monkey anytime soon, such that a call is not appropriate. Again, such is not to argue that the vervet calls, or, for example, those of Diana monkeys in which the severity of an alarm call can be tempered by its use in combination with another call (Candiotti et al., 2012) are lacking informational content – the existence of content has unequivocally been established. Such communication, however, would not appear to have the highest level of *symbolic* reference. But what exactly is meant by “symbolic reference”?

Deacon (1997) devotes a large part of his book to examining what separates reference – in his words, “the symbolic threshold” – from other levels of meaning, and does so much more elegantly than I can summarize here, particularly as my main objective is *not* to define reference but rather to discuss how the acquisition of symbolic, referential communication in nonhumans may affect the ways in which they process information. For the sake of readers of this paper, my interpretation of “symbolic reference” is that it involves semantic and pragmatic use of noniconic symbols – be they auditory/ vocal, manual, or lexical – to stand for (but not be limited to) items such as physical objects and their attributes, various concepts, relations among these items and concepts, actions that can be done to or with these items, and comments about these items (e.g., relating to past/future/hypothetical issues). The use of the term “level of reference” is, again, my interpretation, and follows the above order, where the simplest level involves symbols for objects and attributes, the next level involves symbols for concepts, etc. I expand upon a few of Deacon’s points that bear repeating.

As noted above, distinctions must be made between associations and reference, and such distinctions are made even more difficult given that all reference begins with associations – not in the sense that reference is built up from many associations, but that the earliest stage of learning about reference begins with learning associations: repeated correlations between the presence of the object or action *x* and hearing the sound “x.” Thus, early label acquisition in children is likely more involved with associations than with actual referential meaning – hence the use of holophrases (use of a single word to indicate a variety of situations) as well as over- and under-generalizations of individual words (e.g., calling all four-legged creatures “doggie”). The very *first* label acquired by some nonhumans that are trained by humans probably is simply the association of sound or other symbol with obtaining a reward (a generalized “gimme”) rather than something containing reference. Even the first few labels are still likely simply associations between some signal and item or particular actions with particular situations. In very young children, such associative learning also occurs and generally persists through about the first 10 months, when production is minimal (e.g., Fenson et al., 1994). That is, during this period, if shown two objects – one perceptually salient and one less so – in the presence of a caretaker who focuses on and labels the less interesting object, children ignore that focus and attach/associate the label they hear to what *they* find most salient (Pruden et al., 2006). As more and more labels are acquired, and more and more associations are made, around 12months something changes, and referential acquisition begins to occur. Social cues – the actions and focus of the person doing the labeling – start to take precedence over temporal contiguity (of label and object) and perceptual salience during acquisition; the child engages in joint attention where the adult and child both focus on *the object the adult is labeling* (see Tomasello and Farrar, 1986), sharing their experience – a communicative, *referential* act – and, by 24months, children will ignore a more attractive item that may also be present (Hollich et al., 2000). Such behavior would not be observed if learning was, as it is at 10months, purely associative (Golinkoff and Hirsch-Pasek, 2006). Too, for children, for example, the connection between an object (a food) and its label (“cookie”) does not become extinguished when use of the label does not frequently result in obtaining the referent, as any caretaker of a toddler will attest. If the connection were mere association, extinguished use would indeed be the case (again, see Deacon, 1997; and, yes, one might argue that intermittent reinforcement might strengthen the association, but generally the intervals involved in such human communication – e.g., up to days – are considerably longer than those used in intermittent reinforcement experiments). Once reference is established, use of the label expands; for example, the label is no longer confined to referring to a specific object (“my red round bouncy thing”) or even a small class of objects (“that which I use to play catch”), but can be used to identify novel instances of the item or material (e.g., a golf ball and a basketball are both recognized as “ball”; “wool” can refer to a scarf, a sweater, or even yarn) – or even used in similes and metaphors (“the moon is a balloon” – with a nod to e.e. cummings). Subjects, be they nonhuman or human, can begin to use symbols to ask for labels for novel items (“What’s this?”), demonstrating an understanding of how symbols relate to one another; furthermore, hierarchical categorical labels are then learned, such that a subject knows from which particular, appropriate subset of labels to respond when asked “What color?” vs. “What shape?” (Pepperberg, 1983, 1990a), and after learning a new hue label, immediately understand its *relation* to the category “color” (and likewise for shapes, materials, numbers, etc.). Capacities for comprehension and production now become equivalent (e.g., Pepperberg, 1987b, 1990a,b; Pepperberg and Gordon, 2005). Once reference is established, subjects can use symbols to answer symbolic questions about characteristics of objects that are not immediately present, and can use symbols to request absent items (Pepperberg, 1988a, 1999). So far, although several studies in birds and apes demonstrate their ability to *plan* for the future (e.g., Kabadayi and Osvath, 2017 and references therein), no evidence yet exists for any capacity to use symbols to *refer* to the future. Thus, differences clearly exist in human vs. nonhuman levels of symbolic reference. Nevertheless, unlike nonhuman communication in the wild, for which humans have, as yet, been unable to unequivocally establish symbolic reference, nonhumans who have been taught human systems have demonstrated such reference. For me, the critical issue is one raised by Premack (1983): the extent to which such reference may affect the cognitive processes of those subjects. In the following sections, I will concentrate on my own research and leave reviews of nonhuman primate and cetacean work to researchers in those fields.

## Symbolic Reference and Cognitive Processing, Mostly with Respect to Grey Parrots

Symbolic reference does not guarantee, but enables, abstract thought. Thus, an individual that can represent an object, an action, an attribute, etc., by a symbol can mentally manipulate that symbol, releasing thought processes from the here-and-now [note that an example of nonsymbolic reference would be the approximate number system (ANS) that provides a sense of quantity; the ANS allows distinguishing, e.g., between “more” vs. “less,” but does not enable representation of exact quantity and thus does not enable actions such as multiplication or division). As noted above, simile and metaphor are possible; actions can be planned, tested, and altered without being physically embodied. Premack (1983) thus argued that nonhumans who learned symbolic reference have an enhanced ability to perform tasks that require abstract thinking. He buttressed these claims with data demonstrating that those of his apes that had acquired such symbolic reference outperformed those that did not. And, it was not only apes that could acquire symbolic reference – as noted above, my Grey parrots not only labeled objects, materials, attributes and requested actions, but one parrot, Alex, also used his labels to request new labels and used sound play to devise new labels (Pepperberg, 1990b; additional data reviewed in Pepperberg, 1999). He understood concepts of relative size, number, and of category (i.e., had categorical labels of “color,” “shape,” and “material” and understood what labels were appropriately subsumed under each; Pepperberg, 1999). Other Grey parrots, particularly one named Griffin, have also acquired symbolic reference and succeeded on various cognitive tasks (see below; e.g., Pepperberg and Nakayama, 2016; Clements et al., 2018), often outperforming subjects lacking symbolic reference. I have previously discussed several experiments from my laboratory that give additional credence to Premack (e.g., reviewed in Pepperberg, 2020a,b, 2021, in press). I summarize the importance of symbolic reference for studying cognitive processes and then briefly review some of these studies here.

In some instances, symbolic reference allows the subject to demonstrate cognitive abilities more easily (e.g., may enable them to acquire certain concepts because it allows them to think abstractly; see below) or simply makes it less difficult for humans to interpret the data. In either case, parrots’ vocal plasticity allows us to evaluate their abilities because they can be tested *via symbolic interspecies communication* (Pepperberg, 1981). Interspecies communication (a) directly states the precise content of questions to be asked – animals need not determine the nature of a question through hundreds (if not thousands) of instances of trial-and-error learning, thus making the task efficient; (b) incorporates research showing that social animals may respond more readily and accurately within an ecologically valid social context (Menzel and Juno, 1985); (c) allows facile data comparisons among species, including humans; (d) is an open, arbitrary, creative code with enormous signal variety, enabling an animal to respond in novel, possibly innovative ways that demonstrate greater competence than required responses of operant paradigms, and allows researchers to examine the exact nature *and* extent of information an animal perceives; (e) allows rigorous testing that avoids expectation cuing: Subjects can be made to choose responses from their *entire* repertoire rather than from a subset relevant only to a particular topic. Interspecies communication *via* symbolic reference may thus more facilely demonstrate nonhumans’ inherent capacities or enables their learning of more complex tasks. I now describe a few of several instances in which symbolic reference has been crucial in determining the extent of cognitive abilities in my Grey parrots. Additional studies have been performed for which symbolic reference has allowed testing and demonstration of competence at a level that would not otherwise have been possible (e.g., Piagetian probabilistic reasoning; Clements et al., 2018; reviewed in Pepperberg, in press), likely because such studies involve the use of symbols as abstract place-markers to assist in tasks requiring memory (note Pailian et al., 2020).

## Concepts of Same-Different

A review of this entire topic is the basis for a separate paper (Pepperberg, 2021), but the central issue is as follows (Pepperberg, 1987a): Same-different is more than identity vs. non-identity or the difference in entropy – that is, in overall randomness – between stimuli sets (e.g., Young and Wasserman, 2001). Rather, it is a task that, according to the stringent criteria of Premack (1983), requires a feature analysis of the objects being compared, recognition that objects can simultaneously exhibit attributes that involve *both* similarity and difference, and the ability to understand which attributes are being targeted based on questions of either similarity *or* difference. Because an appropriate response requires that a subject (a) attend to multiple aspects of two different objects; (b) determine, from a verbal question, whether the response is to be based on sameness *or* difference; (c) determine, from the exemplars, *exactly* what is same or different (i.e., what are their colors/shapes/materials?); and then (d) produce, verbally, the label for the hierarchical category of the appropriate attribute, the task is a clear instance in which symbolic reference is likely critical for success – and one that is failed by subjects lacking such abilities (Premack, 1983). Alex succeeded in this task: he could view any two objects, even if he could not label any of their specific attributes, and produce the labels “color,” “shape” or “mah-mah” (his label for matter) in response to questions of “What’s same?” or “What’s different?”; notably, unlike other subjects, he was not limited merely to choosing between symbols representing *same* or *different* or choosing physically between only two objects that were similar to or different from a sample (Premack, 1983) but had to produce the hierarchical category labels from a repertoire of ~70 labels. He eventually learned to respond “none” appropriately to queries about sets that were identical or completely different but only after succeeding on the initial task (Pepperberg, 1988b). By learning symbols – “same”-“different” – to represent the *relations* of categorical commonality – or lack thereof – for specific object pairs, Alex, when experiencing a novel instantiation, could likewise understand *its* relationship to the abstract representation of same-different relationships – as when, queried for the first time “What color bigger?” for two equally-sized items, he asked “What’s same?” (see below, Pepperberg and Brezinsky, 1991). Such fluid response ability requires symbolic, referential, and interspecies communication.

## Relational Concepts: Bigger/Smaller

Understanding relative concepts (darker than, bigger than, etc.) is a more complex task than learning to respond to an absolute concept (e.g., redness; see discussions in Schusterman and Krieger, 1986; Pepperberg and Brezinsky, 1991); it requires a subject to *compare* stimulus choices and then derive and use an underlying, more abstract (and thus general) concept. For example, learning an absolute stimulus value requires a subject to form only a single association (e.g., choose gray; Thomas, 1980), whereas in a task such as “lighter than,” the subject must recognize that what is correct in one trial (“gray” in a task pitting black against gray) may be the incorrect in the next (pitting white against gray). In many tasks, subjects can acquire both absolute and relative knowledge, and because absolute knowledge is acquired more easily, the challenge to an experimenter is to demonstrate whether relative knowledge has also been acquired. Even more difficult is the simultaneous demonstration of both dimensions of relational knowledge – e.g., lighter *and* darker, bigger *and* smaller, same *and* different. A subject that uses symbolic reference, however, can simultaneously be taught labels for both concepts being tested (note Rattermann and Gentner, 1998), rather than having to derive one concept over large numbers of trials (i.e., by being rewarded for choosing only the larger) and then the other through large numbers of reversals (i.e., now being rewarded for choosing only the smaller – in this paradigm, however, *both* concepts may actually *never* be acquired, in that a subject without symbolic reference may simply learn “choose X” vs. “avoid X”; see Hochmann et al., 2016, 2018 for a discussion). Alex, after learning to respond to “What color bigger/smaller?” for three sets of items, transferred, without additional training, to a large number of sets involving sizes outside the training paradigm and to totally novel objects with respect to shape, color, and material; he also spontaneously transferred to “What matter bigger/smaller?” and, when the two objects were equal in size, spontaneously responded “none,” transferring his understanding of that label from the aforementioned study on a lack of same/difference (Pepperberg, 1987a, 1988b; Pepperberg and Brezinsky, 1991). He not only responded to the largest *or* the smallest item present but also recognized that on *any* trial, *either* bigger or smaller could be queried. Such abilities are thus most clearly tested through interspecies communication systems.

## Number Concepts

Almost every living creature that has been studied has demonstrated some sense of number – exact quantification for sets up to 3; approximate quantification for larger sets, for example, “more” vs. “less.” In nonhumans, such abilities have been shown in creatures from fish (Petrazzini et al., 2015) to bears (Vonk and Beran, 2012); in humans, such abilities are found even in preverbal children (Wynn, 1990) and preliterate hunter-gatherer societies (e.g., Frank et al., 2008). However, subjects that understand symbolic reference can go far beyond approximation. They know that a set of *x* elements has precisely *x*, not “about *x*, ±1 or 2.” That is, they can learn that individual symbols represent exact, specific quantities, whatever the items involved: a group of six ants or six elephants or six grapes have different sizes, shapes, masses, etc., but have the same number of elements. Such abilities were once thought to be limited only to humans (reviewed in Pepperberg and Carey, 2012), but a very few nonhumans have demonstrated such exact symbolic number representation, at least for quantities ≤9: two apes, Matsuzawa’s Ai (Matsuzawa, 1985) and Boysen’s Sheba (Boysen and Berntson, 1989), and my subject, the Grey parrot, Alex (Pepperberg, 1987b, 1994).

As I will argue in this section, symbolic reference, importantly, is a prerequisite for advanced number abilities. Although no nonhuman has, as far as we know, invented symbolic numerical representation, those that have acquired such understanding are capable of true counting and simple arithmetic capacities; they can deduce or, at the very least, *learn* cardinality and ordinality and match abilities of ~5-year-old children. I discuss Alex’s abilities in detail with some references to the nonhuman primate research; a full review of the ape studies is beyond the scope of this paper and can be found in papers by Boysen, Matsuzawa, and their students.

True counting, as defined by the several counting principles (“CP,” Gallistel and Gelman, 1992) is not easily acquired. CP state that numerals must be applied in order to items in a set to be enumerated and in a 1–1 correspondence, that the last numeral in a count represents a set’s cardinal value, and that the successor function (that each numeral is known to be exactly one more than the one before it and exactly one less than the one after it; e.g., Carey, 2009) must be understood. This last induction allows for a “bootstrapping” process initially seen only in children. Specifically, the process by which children learn their first few numbers (1–4) is extremely slow (i.e., proceeds over the course of several years), during which time they also simultaneously learn a number line – they learn to state their numerals in a specific order – even though initially the line may make little sense and the order in which they recite their numerals can be variable (Siegel, 1982; Fuson, 1988). Eventually, the ordering of their numerals stabilizes as they learn the *symbolic* meaning of the smaller numerals and they acquire the successor function – and then the bootstrapping process engages: without any further instruction they can now immediately encode the cardinal value expressed by *any* numeral in their now stable count list; the long process used for acquiring the earlier numbers is no longer necessary. In contrast, no nonhuman had shown savings in learning as the successive numerals 5, 6, 7, etc., were added to their repertoire – that is, none had apparently induced the successor function, until Alex (see below). Interestingly, however, Alex did not learn his numerals in order (Pepperberg, 1987b), and all his labels were vocal – meaning that he had to learn not simply to point to a numeral as did the other nonhumans, but rather learn to configure his vocal tract to produce novel utterances (e.g., imagine trying to produce the /v/ sound without lips; see Patterson and Pepperberg, 1998).

Alex nevertheless acquired the ability to use his vocal English labels to quantify sets of one through six objects exactly (i.e., his accuracy did not decrease as the size of the set increased as in the case of the ANS) and was equally accurate when asked to examine novel sets and sets placed in random arrays (Pepperberg, 1987b, 1994). Such behavior is not possible without the use of symbolic reference (Pepperberg, 2020a). Furthermore, Alex, without training, was also able to quantify subsets in a heterogeneous array: given four groups of items that varied in two colors and two object categories (e.g., blue and red keys and trucks), he was able to label the number of items uniquely defined by the conjunction of one color and one object category (e.g., “How many blue key?”) with an accuracy >80% (Pepperberg, 1994).

Interestingly, production and comprehension of number labels may proceed independently. Even young children who are quite proficient at producing the correct number label when asked to quantify a set often fail tests to determine how well they comprehend those labels – if given a bowl of marbles and asked to “Take four,” they often grab a handful rather than the correct amount (Wynn, 1990, 1992). Unlike the other nonhuman subjects, Alex was never trained on number comprehension; nevertheless, when tested, his comprehension accuracy was somewhat superior to that of production (Pepperberg and Gordon, 2005). Again, such abilities are based on symbolic reference.

Alex also acquired other numerical capacities based on symbolic reference. After being trained to identify colored plastic Arabic numerals (1 through 6) – in the absence of any sets of items – with the appropriate vocal labels used for the corresponding numerical sets, he inferred their ordinality by responding to questions of “What color (is the) number (that is) bigger/smaller?” (Pepperberg, 2006b). He again differed from nonhuman primates, who required hundreds of training trials to demonstrate this ability. He acquired a zero-like concept; however, unlike the nonhuman primates (again), he was not trained on the concept but developed it spontaneously, using the previously acquired label “none” (Pepperberg and Gordon, 2005). Like the nonhuman primates, he also spontaneously demonstrated the ability to sum sets of small quantities and label those sets, as well as the ability to provide the label for the sum represented by the combination of Arabic numerals (i.e., recognizing the quantities these abstract symbols represented, combining those quantities, and then representing their sum as a vocal label; Pepperberg, 2006a, 2012). Moreover, after learning the labels for two additional Arabic numerals (7 and 8) in the absence of any sets of items, and their ordinal relationship to previously acquired numerals he, unlike any other nonhuman but like young children, demonstrated the ability to infer their exact cardinality (Pepperberg and Carey, 2012; for a review, see also Pepperberg, 2020b).

## Optical Illusions

How do nonhumans actually *see* the world? Visual systems of most nonhumans, other than those of nonhuman primates, differ considerably from that of humans – for example, nonhumans may have much less or significantly greater color vision, or have much less or greater visual acuity, than humans; they may lack binocular overlap; their neurological architecture may be strikingly different. What exactly are the perceptual processes that are shared across species? We expect that similar evolutionary demands – visual environments, survival needs – may have led to analogous, if not necessarily homologous, solutions concerning some forms of visual processing. Parrots with a repertoire of multiple vocal responses can be rigorously tested for visual competencies, an option yet to be tried in other experimental animals (Pepperberg et al., 2008; Pepperberg and Nakayama, 2016). Specifically, the types of tasks typically used for evaluating human abilities – direct questioning about exactly what is seen – are often unsuited for research with nonverbal species, and thus direct comparisons of nonhumans with humans are not possible. Intensive training procedures were generally necessary to enable nonhumans to discriminate the initial stimulus used in visual tasks, and subjects were then tested on their recognition of similar patterns. Results thus often depended on, for example, statistical averaging over hundreds of trials of pecking/touching behavior to a very limited set of choices, and as a consequence was often highly variable and dependent upon details of the experimental design (Pepperberg et al., 2008). Nonhumans that understand symbolic reference, however, are the exception: those such as Alex and Griffin, who directly communicate with humans and can respond to the exact same stimuli as humans with the exact same responses, thus provide a unique opportunity to state exactly what they see in exactly the same way as do humans.

Given that the avian visual system is notably anatomically and neurobiologically distinct from that of humans (see review in Shimizu et al., 2010 for both similarities and differences), how might a parrot respond to common optical illusions and related visual tasks? These tasks employ early and mid-level vision, and despite neuroanatomical differences, we might expect birds and humans to respond similarly. However, data from experiments using standard operant techniques on some avian subjects were sometimes contradictory and often subject to a variety of alternative interpretations (reviewed in Regolin and Vallortigara, 1995; Pepperberg et al., 2008; Pepperberg and Nakayama, 2016). For example, subjects in these studies may have responded with respect to local cues, mass/number, or stimulus generalization (e.g., Nagasaka et al., 2007).

**Figure 1:**
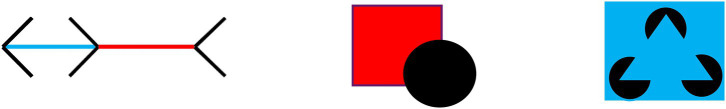
Left to right: Brentano version of ML illusion, occluded figure, Kanizsa figure.

Interestingly, when Alex was tested on the Brentano version of the Müller-Lyer illusion and Griffin tested on amodal and modal completion (respectively, the identity of occluded and illusory Kanizsa figures), by asking them directly what they saw, they responded as did humans. The two horizontal lines in the Brentano figure were of differing colors, and Alex was asked “What color bigger/smaller?”; Griffin was shown standard colored polygons with a black circle covering one corner for amodal completion and shown black pac-men on a colored ground for illusory figures (modal completion) and in both cases asked “What shape is color-X?” (see Figure 1).

For the Müller-Lyer illusion, symbolic reference may merely have allowed facile testing, but for the modal and amodal tests, symbolic reference was likely a necessary factor in Griffin’s ability to respond appropriately. A parrot that understands that a vocal label can represent an item, object, or action is likely to understand the representative relationship between two- and three-dimensional situations. Griffin had learned labels for shapes, and thus that a vocal label could represent an object; he could then understand how two symbols (e.g., one vocal and one visual), which separately represent the same object, can then represent each other (a form of equivalence; Pepperberg, 2006b) and thus how, for example, a three-dimensional entity can be represented by a two-dimensional drawing. That is, he could in turn deduce that the two-dimensional figures represented three-dimensional objects – one item superimposed on another (amodal) or on multiple (modal) items – so that he could appropriately identify pictures of occluded objects and Kanizsa figures (Pepperberg and Nakayama, 2016). The results of these studies (Pepperberg et al., 2008; Pepperberg and Nakayama, 2016) demonstrate how testing nonhumans that understand and appropriately use symbolic reference allows the closest possible comparison with human data, an examination of exactly how nonhumans perceive their world, and of how this perceived information is processed.

## Conclusion

It would seem that each species has its own system for communication, each with its own unique elements and structures, that is, sufficient for its needs in nature. The human system is summarily called “language,” although it, too, consists of a large number of disparate entities, each again having unique elements and structures, including those, for example, based on sign (American Sign Language, British Sign Language, etc.). One can find differences and draw parallels among the various human and nonhuman systems; doing so helps to delineate their various characteristics. The problem with doing so is that humans – despite all of our detailed work in the field for decades – are still fairly incompetent in their ability to perform *complete* analyses of any systems other than their own, so that additional complexities in nonhumans’ systems – and thus possible additional differences and parallels with the human system – currently remain undiscovered, particularly with respect to reference (see Prat, 2019). For example, when birdsongs that are recorded at normal speed are played back at much slower speeds, many small structural differences can be observed among supposedly identical elements, emitted from different songsters or even from the same individual. Are these differences that are indistinguishable to the human ear just a bit of random noise in the system or might they carry important information to an avian listener? Humans do not yet know.

A different, although also only partially successful, tactic in examining nonhuman abilities has been to examine the extent to which nonhumans can acquire the elements of human systems – ASL, vocal labels, artificial systems built on plastic symbols or computer-based lexigrams. As noted above, the underlying premise was that such behavior could not be instilled *de novo*, but that it could be taught only if it were based on some already existent abilities (or even predispositions), such that the extent of success in instilling symbolic reference would provide some evidence for, at least, some cognitive underpinnings of referential nonhuman communication systems. At the time these studies were abruptly ended or their focus shifted (Pepperberg, 2017), no nonhuman had acquired levels of communication equivalent to those of adult humans. However, many of the nonhumans in these studies had acquired symbolic reference and, in many cases, some understanding of very simple combinatory rules for the use of these symbols. The issue of whether nonhumans understand and use such combinations – i.e., can acquire something resembling syntax – is also central for comparing human and nonhuman communication systems. However, what actually constitutes human syntax is another thorny issue, and what some researchers claim are required aspects have been shown to be lacking in some human languages (e.g., Everett, 2005). Thus, I have focused here on the symbols themselves, rather than any hierarchical organization. For a brief review of the importance of combinatorial rules in nature and those acquired by trained nonhumans, as well as their relationship to human syntax, see references cited earlier as well as additional studies and reviews such as ten Cate and Okanoya (2012), Jiang et al. (2018), and Pepperberg (in press).

The point I am trying to make is that the process of understanding that an abstract symbol can represent a concrete *item* may allow a subject to take the next step in understanding that such a symbol can also represent a *concept*, and thereby enable the subject to transfer its knowledge more easily between and among various domains. Once a subject understands that a symbol can represent a concept, the subject can mentally manipulate that symbol, releasing thought processes from the here-and-now. The subject understands how the symbols – and the concepts – are interrelated, such that they immediately understand how to use novel symbols. For example, understanding that some symbols refer to places and others to objects (i.e., representing some conceptual understanding rather than simple associations with concrete items), and that other (even somewhat similar) symbols, such as “want” vs. “wanna go” represent different classes of actions, subjects like Alex know how to use novel combinations appropriately (“I want cracker,” “Wanna go shoulder”) and which to avoid (“Wanna go cracker”) without overt practice (note Leijnen, 2012). Subjects, such as Alex, can also apply the *concept* across domains, understanding, for example, that same-different, even though taught with respect to color/shape/material, will apply to size. Clearly, a few nonhumans, appropriately trained, have demonstrated such abilities; according to Premack (1983), these abilities are exclusively limited to those subjects receiving such training. Of course, in many instances, administering the tasks that demonstrate such abilities would be exceptionally difficult without the use of interspecies symbolic communication; thus, the extent to which such symbolic understanding is *the* critical factor enabling success is possibly still a matter for further study. However, some fairly recent studies comparing adults, young children, and nonhumans suggest that acquisition of symbolic reference – here, the labels “same” and “different” – appears to be a crucial step for being able to solve relational match-to-sample tasks (Hochmann et al., 2017). For example, some studies on *same*/*different* used single arrays having various mixtures of same/different icons: In some, all objects were identical; in some, all different; but for many, ratios of identical to nonidentical objects varied (e.g., Set A: 10 exemplars of one type, three of another, two of a third, and one of a fourth; Set B: four each of four different items). Unlike adult humans, who mostly responded “different” if at least one object differed from all the others – that is, by recognizing same–different relations among individual items within sets – nonhumans and 3-year-old children responded based on ratios of differing elements – on entropy, the array’s overall randomness. Only when children reached about 4years of age and began to use labels “same” and “different” appropriately did they start to respond more like adults on these types of tasks – that is, when they could rely on representations of relations among the various elements in the array; even 5-year olds were below ceiling (Hochmann et al., 2017). Thus, symbolic representation appears necessary for some forms of conceptual knowledge.

Interestingly, the comparative studies of Premack (1983) on the effects of symbolic reference solely involved chimpanzees, a species that lacks vocal learning. Might the absence of that capacity somehow be important with respect to the extent to which symbolic representation affects cognitive processing? Or might the capacity not simply for vocal learning but also for *allospecific* vocal learning be a crucial factor, because allospecific learning implies the ability to transfer concepts across systems and rapidly expand the repertoire (see Deacon, 2012)? We now know that parrots have cortical-like areas that are exceptionally large and more densely packed with neurons than those of nonhuman primates of comparable size (e.g., Jarvis et al., 2013; Olkowicz et al., 2016); that they have specific brain areas and neural connections that support extensive vocal learning – areas that appear less developed in other avian species (including parrots such as keas) that do not engage in allospecific vocal learning, and that are nonexistent in nonhuman species that lack any significant vocal learning – and that these areas also purportedly can be used to expand their intelligence (Chakraborty et al., 2015; Gutiérrez-Ibáñez et al., 2018; again, note Deacon, 2012), particularly with respect to executive function (Herculano-Houzel, 2020). Executive function involves cognitive flexibility, creative problem-solving, reasoning, and mentally representing/relating ideas and facts. Might it thus be possible that some level of reference exists in the communication systems of specific parrot species in nature? So far, evidence is lacking in other vocal learners such as songbirds for anything more than the same form of indexical reference as seen in nonhuman primates (see Beecher, this collection); in parrots, however, a possible system of individual “naming” has been discovered (Berg et al., 2012). Detailed examination of parrot repertoires is still in its infancy compared to the level of examination to which those of nonhuman primates and songbirds have been subjected; most such studies so far have done little other than describe and categorize aggressive, affiliative, and contact calls (e.g., May, 2004; Negrão de Moura et al., 2011).

Clearly, much remains to be studied about nonhuman communication systems, both in the laboratory and in the wild. Might Premack (1983) be correct about the need for symbolic reference in order to succeed on specific cognitive tasks? Or might some level of symbolic reference exist even in untrained nonhumans, providing some evolutionary communicative precursors to human language? If so, the human-nonhuman divide may not be as great as is currently imagined.

## Author Contributions

The author confirms being the sole contributor of this work and has approved it for publication.

## Funding

This research was supported by donors to *The Alex Foundation* and fellowships and NSF grants cited in the referenced journal articles.

## Conflict of Interest

The author declares that the research was conducted in the absence of any commercial or financial relationships that could be construed as a potential conflict of interest.

## Publisher’s Note

All claims expressed in this article are solely those of the authors and do not necessarily represent those of their affiliated organizations, or those of the publisher, the editors and the reviewers. Any product that may be evaluated in this article, or claim that may be made by its manufacturer, is not guaranteed or endorsed by the publisher.

## References

[ref1] Adret-Hausberger M. Güttinger H.-R. Merkel F. W. (1990). Individual life history and song repertoire changes in a colony of starlings (*Sturnus vulgaris*). Ethology 84, 265–280. doi: 10.1111/j.1439-0310.1990.tb00802.x

[ref2] Bandura A. (ed.) (1971). “Analysis of modeling processes,” in Psychological Modeling (Chicago: Aldine-Athcrton), 1–62.

[ref3] Baptista L. F. Petrinovich L. (1984). Social interaction, sensitive phases, and the song template hypothesis in the white-crowned sparrow. Anim. Behav. 32, 172–181. doi: 10.1016/S0003-3472(84)80335-8

[ref4] Baptista L. F. Petrinovich L. (1986). Song development in the white-crowned sparrow: social factors and sex differences. Anim. Behav. 34, 1359–1371. doi: 10.1016/S0003-3472(86)80207-X

[ref5] Beecher M. D. Brenowitz E. A. (2005). Functional aspects of song learning in the songbirds. Trends Ecol. Evol. 20, 143–149. doi: 10.1016/j.tree.2005.01.004, PMID: 16701358

[ref6] Beecher M. D. Campbell E. Burt J. M. Hill C. E. Nordby J. C. (2000). Song-type matching between neighbouring song sparrows. Anim. Behav. 59, 21–27. doi: 10.1006/anbe.1999.1276, PMID: 10640363

[ref7] Berg K. S. Delgado S. Cortopassi K. A. Bessinger S. R. Bradbury J. W. (2012). Vertical transmission of learned signatures in a wild parrot. Proc. Biol. Sci. 279, 585–591. doi: 10.1098/rspb.2011.0932, PMID: 21752824 PMC3234552

[ref8] Berwick R. C. Beckers G. J. L. Okanoya K. Bolhuis J. J. (2012). A bird’s eye view of human language evolution. Front. Evol. Neurosci. 4:5. doi: 10.3389/fnevo.2012.00005, PMID: 22518103 PMC3325485

[ref9] Bickerton D. (1990). Language and Species. Chicago: University of Chicago Press.

[ref10] Boysen S. T. Berntson G. G. (1989). Numerical competence in a chimpanzee (*Pan troglodytes*). J. Comp. Psychol. 103, 23–31. doi: 10.1037/0735-7036.103.1.23, PMID: 2924529

[ref11] Bronowski J. Bellugi U. (1970). Language, name and concept. Science 168, 669–673. doi: 10.1126/science.168.3932.669, PMID: 5438497

[ref12] Busnel R. G. Mebes H. D. (1975). Hearing and communication in birds: the cocktail party effect in intraspecific communication of *Agapornis roseicollis*. Life Sci. 17, 1567–1569. doi: 10.1016/0024-3205(75)90178-2, PMID: 1207375

[ref13] Byers B. (1995). Song types, repertoires and song variability in a population of chestnut-sided warblers. Condor 97, 390–401. doi: 10.2307/1369025

[ref14] Candiotti A. Zuberbühler K. Lemasson A. (2012). Context-related call combinations in female Diana monkeys. Anim. Cogn. 15, 327–339. doi: 10.1007/s10071-011-0456-8, PMID: 21947942

[ref310] Carey S. (2009). The Origin of Concepts. New York: Oxford University Press.

[ref15] Catchpole C. K. (1983). Variation in the song of the great reed warbler *Acrocephalus arundinaceus* in relation to mate attraction and territorial defence. Anim. Behav. 3, 1217–1225.

[ref16] Chakraborty M. Walløe S. Nedergaard S. Fridel E. E. Dabelsteen T. Pakkenberg B.. (2015). Core and shell song systems unique to the parrot brain. PLoS One 10:e0118496. doi: 10.1371/journal.pone.0118496, PMID: 26107173 PMC4479475

[ref17] Cheney D. L. Seyfarth R. M. (1985). Social and nonsocial knowledge in vervet monkeys. Philos. Trans. R. Soc. B 308, 187–201. doi: 10.1098/rstb.1985.0019

[ref18] Clements K. Gray S. L. Gross B. Pepperberg I. M. (2018). Initial evidence for probabilistic learning by a Grey parrot (*Psittacus erithacus*). J. Comp. Psychol. 132, 166–177. doi: 10.1037/com0000106, PMID: 29528667

[ref19] Cobb S. (1960). “Observations on the comparative anatomy of the avian brain,” in Perspectives in Biology and Medicine. Vol. 3. eds. Ingle D. I. Waife S. O. (Chicago: University of Chicago Press), 383–408. 10.1353/pbm.1960.0053 13810776

[ref20] Collier K. Bickel B. van Schaik C. P. Manser M. B. Townsend S. W. (2014). Language evolution: syntax before phonology? Proc. R. Soc. B 281:20140263. doi: 10.1098/rspb.2014.0263, PMID: 24943364 PMC4083781

[ref21] Colquitt B. M. Merullo D. P. Konopka G. Roberts T. F. Brainard M. S. (2021). Cellular transcriptomics reveals evolutionary identities of songbird vocal circuits. Science 371:695. doi: 10.1126/science.abd9704 PMC8136249 33574185

[ref22] Darwin C. (1871). The Descent of Man and Selection in Relation to Sex. Vol. 2. London: John Murray.

[ref23] de Boysson-Bardies B. Halle P. Sagart L. Durand C. (1989). A crosslinguistic investigation of vowel formants in babbling. J. Child Lang. 16, 1–17. doi: 10.1017/S0305000900013404, PMID: 2925806

[ref24] Deacon T. W. (1997). The Symbolic Species: The Co-evolution of Language and the Brain. New York: W.W. Norton & Company, Inc.

[ref25] Deacon T. W. (2012). “Beyond the symbolic species,” in The Symbolic Species Evolved. eds. Schilhab T. Stjernfelt F. Deacon T. (Heidelberg, London, New York: Springer), 9–38.

[ref26] Dilger W. C. (1956). Hostile behavior and reproductive isolating mechanisms in the avian genera *Catharus* and *Hylocichla*. Auk 73, 313–353. doi: 10.2307/4082003

[ref27] Doupe A. J. Kuhl P. K. (1999). Birdsong and human speech: common themes and mechanisms. Ann. Rev. Neurosci. 22, 567–631. doi: 10.1146/annurev.neuro.22.1.567, PMID: 10202549

[ref28] Everett D. L. (2005). Cultural constraints on grammar and cognition in the Pirahã: another look at the design features of human language. Curr. Anthropol. 46, 621–646. doi: 10.1086/431525

[ref29] Fenson L. Dale P. Reznick S. Bates E. Thal D. Pethick S. (1994). Variability in early communicative development. Monogr. Soc. Res. Child Dev. 59, 1–173. PMID: 7845413

[ref30] Fischer J. (2011). “Where is the information in animal communication?” in Animal Thinking: Contemporary Issues in Comparative Cognition. eds. Menzel R. Fischer J. (Cambridge, MA: MIT Press), 151–161.

[ref31] Frank M. C. Everett D. L. Fedorenko E. Gibson E. (2008). Number as a cognitive technology: evidence from Pirahã language and cognition. Cognition 108, 819–824. doi: 10.1016/j.cognition.2008.04.007, PMID: 18547557

[ref32] Fromkin V. Krashen S. Curtiss S. Rigler D. Rigler M. (1974). Development of language in genie: case of language acquisition beyond the ‘critical period’. Brain Lang. 1, 81–107. doi: 10.1016/0093-934X(74)90027-3

[ref33] Fuson K. (1988). Children’s Counting and Concepts of Number. New York: Springer-Verlag.

[ref34] Gallistel C. R. Gelman R. (1992). Preverbal and verbal counting and computation. Cognition 44, 43–74. doi: 10.1016/0010-0277(92)90050-R, PMID: 1511586

[ref35] Gardner R. A. Gardner B. T. (1969). Teaching sign language to a chimpanzee. Science 187, 644–672. doi: 10.1126/science.165.3894.664, PMID: 5793972

[ref36] Golinkoff R. M. Hirsch-Pasek K. (2006). Baby wordsmith. Curr. Dir. Psychol. Sci. 15, 30–33. doi: 10.1111/j.0963-7214.2006.00401.x

[ref37] Griffin D. R. (1976). The Question of Animal Awareness. New York: Rockefeller University Press.

[ref38] Grimshaw G. M. Adelstein A. Bryden M. P. MacKinnon G. E. (1998). First-language acquisition in adolescence: evidence for a critical period for verbal language development. Brain Lang. 63, 237–255. doi: 10.1006/brln.1997.1943, PMID: 9654433

[ref39] Grosslight J. H. Zaynor W. C. (1967). “Vocal behavior of the mynah bird,” in Research in Verbal Behavior and Some Neurophysiological Implications. eds. Salzinger K. Salzinger S. (New York: Academic Press), 5–9.

[ref40] Gutiérrez-Ibáñez C. Iwaniuk A. N. Wylie D. R. (2018). Parrots have evolved a primate-like telencephalic-midbrain-cerebellar circuit. Sci. Rep. 8:9960. doi: 10.1038/s41598-018-28301-4, PMID: 29967361 PMC6028647

[ref330] Hailman J. Elowson M. (1984). Personal Communication.

[ref41] Hartshorne J. K. Tennebaum J. B. Pinker S. (2018). A critical period for second language acquisition: evidence from 2/3 million English speakers. Cognition 177, 263–277. doi: 10.1016/j.cognition.2018.04.007, PMID: 29729947 PMC6559801

[ref42] Hayes K. J. Nissen C. H. (1971). “Higher mental functions of a home-raised chimpanzee,” in Behavior of Nonhuman Primates. Vol. 4. eds. Schrier A. M. Stollnitz F. (New York, NY: Academic Press) (Original work published in 1956), 57–115.

[ref43] Hedges S. B. Parker P. H. Sibley C. G. Kumar S. (1996). Continental breakup and the ordinal diversification of birds and mammals. Nature 381, 226–229. doi: 10.1038/381226a0, PMID: 8622763

[ref44] Herculano-Houzel S. (2020). Birds do have a brain cortex—and think. Science 369, 1567–1568. doi: 10.1126/science.abe0536, PMID: 32973020

[ref45] Herman L. M. (ed.) (1980). “Cognitive characteristics of dolphins,” in Cetacean Behavior (New York, NY: Wiley), 408–409.

[ref46] Hochmann J.-R. Carey S. Mehler J. (2018). Infants learn a rule predicated on the relation same but fail to simultaneously learn a rule predicated on the relation different. Cognition 177, 49–57. doi: 10.1016/j.cognition.2018.04.005, PMID: 29635110

[ref47] Hochmann J.-R. Mody S. Carey S. (2016). Infants’ representations of same and different in match- and non-match-to-sample tasks. Cogn. Psychol. 86, 87–811. doi: 10.1016/j.cogpsych.2016.01.005, PMID: 26970605 PMC4935928

[ref48] Hochmann J.-R. Tuerk A. S. Sandborn S. Zhu R. Long R. Dempster M.. (2017). Children’s representation of abstract relations in relational/array match-to-sample tasks. Cogn. Psychol. 99, 17–43. doi: 10.1016/j.cogpsych.2017.11.001, PMID: 29132016

[ref49] Hockett C. F. Altmann S. A. (1968). “A note on design features,” in Animal Communication: Techniques of Study and Results of Research. ed. Sebeok T. (Bloomington, IN: Indiana University Press), 61–72.

[ref50] Hollich G. J. Hirsh-Pasek K. Golinkoff R. M. Brand R. J. Brown E. Chung H. L.. (2000). Breaking the language barrier: An emergentist coalition model for the origins of word learning. Monog. Soc. Res. Child Dev. 65, i–vi. 12467096

[ref320] Howard E. (1920). Territory and Bird Life. Glasgow, Scotland, UK: Williams Collins Sons & Company, Limited.

[ref51] Jarvis E. D. (2019). Evolution of vocal learning and spoken language. Science 366, 50–54. doi: 10.1126/science.aax0287, PMID: 31604300

[ref52] Jarvis E. D. Güntürkün O. Bruce L. Csillag A. Karten H. Kuenzel W.. (2005). Avian brains and a new understanding of vertebrate brain evolution. Nat. Rev. Neurosci. 6, 151–159. doi: 10.1038/nrn1606, PMID: 15685220 PMC2507884

[ref53] Jarvis E. D. Yu J. Rivas M. V. Horita H. Feenders G. Whitney O.. (2013). Global view of the functional molecular organizations of the avian cerebrum: mirror images and functional columns. J. Comp. Neurol. 521, 3614–3665. doi: 10.1002/cne.23404, PMID: 23818122 PMC4145244

[ref54] Jiang X. Long T. Cao W. Li J. Dehaene S. Wang L. (2018). Production of supraregular spatial sequences by macaque monkeys. Curr. Biol. 28, 1851–1859. doi: 10.1016/j.cub.2018.04.047, PMID: 29887304 PMC6606444

[ref55] Johnson J. S. Newport E. L. (1989). Critical period effects in second language learning: the influence of maturational state on the acquisition of English as a second language. Cogn. Psychol. 21, 60–99. doi: 10.1016/0010-0285(89)90003-0, PMID: 2920538

[ref56] Kabadayi C. Osvath M. (2017). Ravens parallel great apes in flexible planning for tool-use and bartering. Science 357, 202–204. doi: 10.1126/science.aam8138, PMID: 28706072

[ref57] Kellogg W. N. (1968). Communication and language in the home-raised chimpanzee. Science 162, 423–438. doi: 10.1126/science.162.3852.423, PMID: 5683047

[ref58] Konishi M. (1964). Song variation in a population of Oregon juncos. Condor 66, 423–436. doi: 10.2307/1365432

[ref59] Krebs J. R. (1977). “Song and territory in the great tit *Parus major,*” in Evolutionary Ecology. eds. Stonehouse B. Perrins C. (London: Macmillan), 47–62.

[ref60] Krebs J. R. Ashcroft R. Webber M. I. (1978). Song repertoires and territory defense in the great tit. Nature 271, 539–542. doi: 10.1038/271539a0

[ref61] Kroodsma D. E. (1976). Reproductive development in a female songbird: differential stimulation by quality of male song. Science 192, 574–575. doi: 10.1126/science.192.4239.574, PMID: 17745657

[ref62] Kroodsma D. E. Bereson R. C. Byers B. E. Minear E. (1989). Use of song types by the chestnut-sided warbler: evidence for both intra- and inter-sexual functions. Can. J. Zool. 67, 447–456. doi: 10.1139/z89-065

[ref63] Lachman R. Mister-Lachman J. (1974). Language in man, monkeys, and machines. Science 185, 871–873. doi: 10.1126/science.185.4154.871, PMID: 11785524

[ref64] Lawson R. W. Lanning D. V. (1980). “Nesting and status of the maroon-fronted parrot (*Rhynchopsitta terrisi*),” in Conservation of New World Parrots. ed. Pasquier R. F. (Washington, DC: ICBP Tech. Publ. No. 1), 385–392.

[ref65] Leijnen S. (2012). “Emerging symbols,” in The Symbolic Species Evolved. eds. Schilhab T. Stjernfelt F. Deacon T. (Heidelberg, London, New York: Springer), 253–262.

[ref66] Lenneberg E. H. (1967). Biological Foundations of Language. New York: Wiley.

[ref67] Levinson S. T. (1980). “The social behavior of the white-fronted Amazon (*Amazona albifrons*),” in Conservation of New World Parrots. ed. Pasquier R. F. (Washington, DC: ICBP Tech. Publ. No. 1), 403–417.

[ref68] Lilly J. C. (1967). “Dolphin’s vocal mimicry as a unique ability and a step toward understanding,” in Research in Verbal Behavior and Some Neurophysiological Implications. eds. Salzinger K. Salzinger S. (New York, NY: Academic Press), 21–27.

[ref69] Lofting H. (1920). The Story of Dr. Doolittle. New York: Frederick A. Stokes.

[ref70] Lorenz K. (1952). King Solomon's Ring. trans. M. K. Wilson (New York: Harper & Row).

[ref71] Marks J. M. (2005). What It Means to Be 98% Chimpanzee: Apes, People, and Their Genes. Berkeley: University of California Press.

[ref72] Marler P. (1952). Variation in the song of the chaffinch, *Fringilla coelebs*. Ibis 94, 458–472. doi: 10.1111/j.1474-919X.1952.tb01845.x

[ref73] Marler P. (1956). The voice of the chaffinch and its function as a language. Ibis 98, 231–261. doi: 10.1111/j.1474-919X.1956.tb03042.x

[ref74] Marler P. (1960). “Bird songs and mate selection,” in Animal Sounds and Communication. ed. Tavolga W. N. (Washington, DC: A.I.B.S. Symposium Proceedings), 348–367.

[ref75] Marler P. (1961). The logical analysis of animal communication. J. Theor. Biol. 1, 295–317. doi: 10.1016/0022-5193(61)90032-7, PMID: 13766984

[ref76] Marler P. (1970a). A comparative approach to vocal learning: song development in white-crowned sparrows. J. Comp. Physiol. Psychol. 71, 1–25. doi: 10.1037/h0029144

[ref77] Marler P. (1970b). Birdsong and speech development: could there be parallels? Am. Sci. 58, 669–673. PMID: 5480089

[ref78] Marler P. (1974). “Animal communication,” in Nonverbal Communication: Advances in the Study of Communication and Affect. eds. Krames L. Pliner P. Alloway T. (New York: Plenum Press), 25–50.

[ref79] Marler P. (1977). “The structure of animal communication sounds,” in Recognition of Complex Acoustic Signals. ed. Bullock T. H. (Berlin: Dahlem Workshop, Abakon-Verlagsgesellschaft), 17–35.

[ref80] Marx J. (1980). Ape-language controversy flares up. Science 207, 1330–1333. doi: 10.1126/science.7355293, PMID: 7355293

[ref81] Matsuzawa T. (1985). Use of numbers by a chimpanzee. Nature 315, 57–59. doi: 10.1038/315057a0, PMID: 3990808

[ref82] May D. (2004). The vocal repertoire of Grey parrots (*Psittacus erithacus*) living in the Congo Basin (Central African Republic, Cameroon). doctoral dissertation. Tucson, AZ: University of Arizona.

[ref83] Menzel E. W. Jr. Juno C. (1985). Social foraging in marmoset monkeys and the question of intelligence. Phil. Trans. R. Soc. London B308, 145–158. doi: 10.1098/rstb.1985.0016

[ref84] Miles H. L. (1978). “Language acquisition in apes and children,” in Sign Language Acquisition in Man and Ape. ed. Peng F. C. C. (Boulder, CO: Westview Press), 108–120.

[ref85] Mowrer O. H. (1950). Learning Theory and Personality Dynamics. New York: Ronald Press.

[ref86] Mowrer O. H. (1952). The autism theory of speech development and some clinical applications. J. Speech Hear. Disord. 17, 263–268. doi: 10.1044/jshd.1703.263 13053547

[ref87] Mowrer O. H. (1954). A psychologist looks at language. Am. Psychol. 9, 660–694. doi: 10.1037/h0062737

[ref88] Nagasaka Y. Lazareva O. F. Wasserman E. A. (2007). Prior experience affects amodal completion in pigeons. Percept. Psychophys. 69, 596–605. doi: 10.3758/BF03193917, PMID: 17727113

[ref89] Nauta W. J. H. Karten H. J. (1971). “A general profile of the vertebrate brain, with sidelights on the ancestry of cerebral cortex,” in The Neurosciences Second Study Program. Evolution of Brain and Behavior. ed. Schmitt F. O. (New York: Rockefeller University Press), 7–26.

[ref90] Negrão de Moura L. da Silva M. L. Vielliard J. (2011). Vocal repertoire of wild breeding orange-winged parrots *Amazona amazonica* in Amazonia. Bioacoustics 20, 331–339. doi: 10.1080/09524622.2011.9753655

[ref91] Nice M. M. (1943). Studies in the Life History of the Song Sparrow, II. New York: Linnaean Society.

[ref92] Nottebohm F. (1966). The role of sensory feedback in the development of avian vocalizations. doctoral dissertation. Berkeley, CA: University of California.

[ref93] Nottebohm F. (1970). Ontogeny of bird song. Science 167, 950–956. doi: 10.1126/science.167.3920.950, PMID: 17749614

[ref94] Nottebohm F. (2006). The road we travelled: discovery, choreography, and significance of brain replaceable neurons. Ann. N. Y. Acad. Sci. 1016, 628–658. doi: 10.1196/annals.1298.027, PMID: 15313798

[ref95] Olkowicz S. Kocourek M. Lucan R. K. Porteš M. Fitch W. T. Herculano-Houzel S.. (2016). Birds have primate-like numbers of neurons in the forebrain. Proc. Natl. Acad. Sci. U. S. A. 113, 7255–7260. doi: 10.1073/pnas.1517131113, PMID: 27298365 PMC4932926

[ref96] Oller D. K. (1978). Infant vocalizations and the development of speech. Allied Health Behav. Sci. 1, 523–549.

[ref97] Oller D. K. Weiman L. A. Doyle W. J. Ross C. (1976). Infant babbling and speech. J. Child Lang. 3, 1–11. doi: 10.1017/S0305000900001276

[ref98] Pailian H. Carey S. Halberta J. Pepperberg I. M. (2020). Age and species comparisons of visual mental manipulation ability as evidence for its development and evolution. Sci. Rep. 10:7689. doi: 10.1038/s41598-020-64666-1, PMID: 32376944 PMC7203154

[ref99] Patterson F. (1978). The gestures of a gorilla: language acquisition in another pongid. Brain Lang. 5, 72–97. doi: 10.1016/0093-934X(78)90008-1, PMID: 618570

[ref100] Patterson D. K. Pepperberg I. M. (1998). A comparative study of human and Grey parrot phonation: II. Acoustic and articulatory correlates of stop consonants. J. Acoust. Soc. Am. 103, 2197–2213. 9566339 10.1121/1.421365

[ref101] Pepperberg I. M. (1981). Functional vocalizations of an African Grey parrot (*Psittacus erithacus*). Z. Tierpsychol. 55, 139–160.

[ref102] Pepperberg I. M. (1983). Cognition in the African Grey parrot: preliminary evidence for auditory/vocal comprehension of the class concept. Anim. Learn. Behav. 11, 179–185. doi: 10.3758/BF03199646

[ref103] Pepperberg I. M. (1987a). Acquisition of the same/different concept by an African Grey parrot (*Psittacus erithacus*): learning with respect to categories of color, shape, and material. Anim. Learn. Behav. 15, 423–432. doi: 10.3758/BF03205051

[ref104] Pepperberg I. M. (1987b). Evidence for conceptual quantitative abilities in the African Grey parrot: labeling of cardinal sets. Ethology 75, 37–61. doi: 10.1111/j.1439-0310.1987.tb00641.x

[ref105] Pepperberg I. M. (1988a). An interactive modeling technique for acquisition of communication skills: separation of ‘labeling’ and ‘requesting’ in a psittacine subject. Appl. Psycholinguist. 9, 59–76. doi: 10.1017/S014271640000045X

[ref106] Pepperberg I. M. (1988b). Acquisition of the concept of absence by an African Grey parrot: learning with respect to questions of same/different. J. Exp. Anal. Behav. 50, 553–564. doi: 10.1901/jeab.1988.50-553, PMID: 16812572 PMC1338917

[ref107] Pepperberg I. M. (1990a). Cognition in an African Grey parrot (*Psittacus erithacus*): further evidence for comprehension of categories and labels. J. Comp. Psychol. 104, 41–52. doi: 10.1037/0735-7036.104.1.41

[ref108] Pepperberg I. M. (1990b). Referential mapping: attaching functional significance to the innovative utterances of an African Grey parrot (*Psittacus erithacus*). Appl. Psycholinguist. 11, 23–44. doi: 10.1017/S0142716400008274

[ref109] Pepperberg I. M. (1994). Evidence for numerical competence in an African Grey parrot (*Psittacus erithacus*). J. Comp. Psychol. 108, 36–44. doi: 10.1037/0735-7036.108.1.36

[ref110] Pepperberg I. M. (1999). The Alex Studies. Cambridge, MA: Harvard University Press.

[ref111] Pepperberg I. M. (2006a). Grey parrot (*Psittacus erithacus*) numerical abilities: addition and further experiments on a zero-like concept. J. Comp. Psychol. 120, 1–11. doi: 10.1037/0735-7036.120.1.1, PMID: 16551159

[ref112] Pepperberg I. M. (2006b). Ordinality and inferential abilities of a Grey parrot (*Psittacus erithacus*). J. Comp. Psychol. 120, 205–216. doi: 10.1037/0735-7036.120.3.205, PMID: 16893258

[ref410] Pepperberg I. M. (2012). Further evidence for addition and numerical competence by a Grey parrot (*Psittacus erithacus*). Anim. Cogn. 15, 711–717. doi: 10.1007/s10071-012-0470-5, PMID: 22402776

[ref113] Pepperberg I. M. (2017). Animal language studies: what happened? Special issue. Psychon. Bull. Rev. 24, 181–185. doi: 10.3758/s13423-016-1101-y 27368639

[ref114] Pepperberg I. M. (2020a). Human-avian comparisons in cognitive performance. Front. Psychol, 11–973. doi: 10.3389/fpsyg.2020.00973

[ref115] Pepperberg I. M. (2020b). “The comparative psychology of intelligence: some thirty years later,” in Special Issue of Frontiers in Psychology: Comparative Psychology. eds. Scarf D. Colombo M.. 10.3389/fpsyg.2020.00973 PMC7248277 32508723

[ref116] Pepperberg I. M. (2020c). Vocal communication in nonhuman animals: view from the wings. Anim. Behav. Cogn. 7, 95–100. doi: 10.26451/abc.07.02.03.2020

[ref117] Pepperberg I. M. (2021). How do a pink plastic flamingo and a pink plastic elephant differ? Evidence for abstract representations of the relations of same-different in a Grey parrot. Curr. Opin. Behav. Sci. 37, 146–152. doi: 10.1016/j.cobeha.2020.12.010

[ref118] Pepperberg I. M. (in press). “Symbolic communication in the Grey parrot,” in Cambridge Handbook of Animal Cognition. eds. Kaufman A. Call J. (Cambridge, UK: Cambridge University Press).

[ref119] Pepperberg I. M. Brezinsky M. V. (1991). Relational learning by an African Grey parrot (*Psittacus erithacus*): discriminations based on relative size. J. Comp. Psychol. 105, 286–294. doi: 10.1037/0735-7036.105.3.286 1935007

[ref120] Pepperberg I. M. Carey S. (2012). Grey parrot number acquisition: the inference of cardinal value from ordinal position on the numeral list. Cognition 125, 219–232. doi: 10.1016/j.cognition.2012.07.003, PMID: 22878117 PMC3434310

[ref121] Pepperberg I. M. Gordon J. D. (2005). Numerical comprehension by a Grey parrot (*Psittacus erithacus*), including a zero-like concept. J. Comp. Psychol. 119, 197–209. doi: 10.1037/0735-7036.119.2.197, PMID: 15982163

[ref122] Pepperberg I. M. Nakayama K. (2016). Robust representation of shape by a Grey parrot (*Psittacus erithacus*). Cognition 153, 146–160. doi: 10.1016/j.cognition.2016.04.014, PMID: 27206312

[ref420] Pepperberg I. M. Vicinay J. Cavanagh P. (2008). The Müller-Lyer illusion is processed by a Grey parrot (*Psittacus erithacus*). Perception 37, 765–781. doi: 10.1068/p5898 18605149

[ref123] Peters S. Nowicki S. (2017). Overproduction and attrition: the fates of songs memorized during song learning in songbirds. Anim. Behav. 124, 255–261. doi: 10.1016/j.anbehav.2016.09.019

[ref124] Petrazzini M. E. M. Agrillo C. Izard V. Bisazza A. (2015). Relative versus absolute numerical representation: can guppies represent “fourness”? Anim. Cogn. 18, 1007–1017. doi: 10.1007/s10071-015-0868-y 25846961

[ref125] Pierce C. S. (1978). Collected Papers. eds. Hartshorne C. Weiss P. (Cambridge, MA: Belknap).

[ref430] Portmann A. Stingelin W. (1961). “The central nervous system” in Biology and Comparative Physiology of Birds. Vol. 2. ed. Marshall A. J. (New York: Academic Press), 1–36.

[ref126] Power D. M. (1966a). Agnostic behavior and vocalizations of orange-chinned parakeets in captivity. Condor 68, 562–558. doi: 10.2307/1366264

[ref127] Power D. M. (1966b). Antiphonal duetting and evidence for auditory reaction time in the orange chinned parakeet. Auk 83, 314–319. doi: 10.2307/4083033

[ref128] Prat Y. (2019). Animals have no language, and humans are animals too. Perspect. Psychol. Sci. 14, 885–893. doi: 10.1177/1745691619858402, PMID: 31398084

[ref129] Premack D. (1971). “On the assessment of language-competence in the chimpanzee,” in Behavior of Nonhuman Primates. Vol. 4. eds. Schrier A. M. Stollnitz F. (New York, NY: Academic Press), 186–228.

[ref130] Premack D. (1983). The codes of man and beast. Behav. Brain Sci. 6, 125–167. doi: 10.1017/S0140525X00015077

[ref131] Price T. Wadewitz P. Cheney D. Seyfarth R. Hammerschmidt K. Fischer J. (2014). Vervets revisited: A quantitative analysis of alarm call structure and context specificity. Sci. Rep. 5:13220. doi: 10.1038/srep13220, PMID: 26286236 PMC4541072

[ref132] Pruden S. M. Hirsh-Pasek K. Golinkoff R. M. Hennon E. A. (2006). The birth of words: ten-month-olds learn words through perceptual salience. Child Dev. 77, 266–280. doi: 10.1111/j.1467-8624.2006.00869.x, PMID: 16611171 PMC4621011

[ref133] Rasmussen K. (1972). “The Netsilik Eskimos,” in Shaking the Pumpkin. ed. Rothenberg J. (Garden City, NY: Doubleday), 45.

[ref134] Rattermann M. J. Gentner D. (1998). “The use of relational labels improves young children’s performance in a mapping task,” in Advances in Analogy Research: Integration of Theory and Data From the Cognitive, Computational, and Neural Sciences. eds. Holyoak K. Gentner D. Kokinov B. (Sophia: New Bulgarian University Press), 274–282.

[ref135] Regolin L. Vallortigara G. (1995). Perception of partly occluded objects in young chicks. Percept. Psychophys. 57, 971–976. doi: 10.3758/BF03205456, PMID: 8532500

[ref136] Reiner A. Perkel D. J. Bruce L. L. Butler A. Csillag A. Kuenzel W.. (2004). Revised nomenclature for avian telencephalon and some related brainstem nuclei. J. Comp. Neurol. 473, 377–414. doi: 10.1002/cne.20118, PMID: 15116397 PMC2518311

[ref137] Rice J. O. Thompson W. L. (1968). Song development in the indigo bunting. Anim. Behav. 16, 462–469. doi: 10.1016/0003-3472(68)90041-9

[ref138] Ridgeway S. H. (1990). “The central nervous system of the bottlenosed dolphin,” in The Bottlenosed Dolphin. eds. Leatherwood S. Reeves R. R. (San Diego: Academic Press), 69–97.

[ref139] Rumbaugh D. M. (1977). Language Learning by a Chimpanzee. New York, NY: Academic Press.

[ref140] Saunders A. A. (1951). The song of the song sparrow. Wils. Bull. 63, 99–109.

[ref530] Schusterman R. J. Krieger K. (1986). Artificial language comprehension and size transposition by a California sea lion (*Zalophus californianus*). J. Comp. Psychol. 100, 348–355. doi: 10.1037/0735-7036.100.4.348 3802779

[ref141] Sebeok T. A. Rosenthal R. (eds.) (1981). The Clever Hans Phenomenon: Communication With Horses, Whales, Apes, and People (New York: New York Academy of Sciences Annals). 6942738

[ref142] Seyfarth R. M. Cheney D. L. Bergman T. Fischer J. Züberbühler K. Hammerschmidt K. (2010). The central importance of information in studies of animal communication. Anim. Behav. 80, 3–8. doi: 10.1016/j.anbehav.2010.04.012

[ref143] Seyfarth R. M. Cheney D. L. Marler P. (1980a). Monkey responses to three different alarm calls: evidence of predator classification and semantic communication. Science 210, 801–803. doi: 10.1126/science.7433999, PMID: 7433999

[ref144] Seyfarth R. M. Cheney D. L. Marler P. (1980b). Vervet monkey alarm calls: semantic communication in a free-ranging primate. Anim. Behav. 28, 1070–1094. doi: 10.1016/S0003-3472(80)80097-2

[ref540] Shimizu T. Patton T. B. Husband S. A. (2010). Avian visual behavior: organization of the telencephalon. Brain Behav. Evol. 75, 204–217. doi: 10.1159/000314283, PMID: 20733296 PMC2977968

[ref145] Siegel L. S. (1982). “The development of quantity concepts: perceptual and linguistic factors,” in Children’s Logical and Mathematical Cognition. ed. Brainerd C. J. (Berlin: Springer-Verlag), 123–155.

[ref146] Smith W. J. (1963). Vocal communication of information in birds. Am. Nat. 97, 117–125. doi: 10.1086/282261

[ref147] Smith W. J. (1996). Information about behavior provided by Louisiana waterthrush, *Seurus motacilla* (Parulinae), songs. Anim. Behav. 51, 785–799. doi: 10.1006/anbe.1996.0083

[ref148] Struhsaker T. T. (1967). “Auditory communication among vervet monkeys (*Cercopithecus aethiops*),” in Social Communication Among Primates. ed. Altmann S. A. (Chicago: University of Chicago Press), 281–324.

[ref149] Suzuki T. N. Griesser M. Wheatcroft D. (2019). Syntactic rules in avian vocal sequences as a window into the evolution of compositionality. Anim. Behav. 151, 267–274. doi: 10.1016/j.anbehav.2019.01.009

[ref150] ten Cate C. Okanoya K. (2012). Revisiting the syntactic abilities of non-human animals: natural vocalizations and artificial grammar learning. Phil. Trans. R. Soc. Lond. B Biol. Sci. 367, 1984–1994. doi: 10.1098/rstb.2012.0055, PMID: 22688634 PMC3367684

[ref151] Terrace H. S. Petitto L. A. Sanders R. J. Bever T. G. (1979). Can an ape create a sentence? Science 206, 891–902. doi: 10.1126/science.504995, PMID: 504995

[ref510] Thomas R. K. (1980). Evolution of intelligence: an approach to its assessment. Brain Behav. Evol. 17, 454–472. doi: 10.1159/000121814, PMID: 7437899

[ref152] Thorpe W. H. (1958). The learning of song patterns by birds, with special reference to the song of the chaffinch, *Fringilla coelebs*. Ibis 100, 535–570.

[ref153] Thorpe W. H. (1974). Animal and Human Nature. New York: Doubleday.

[ref154] Todt D. (1975). Social learning of vocal patterns and modes of their applications in Grey parrots. Z. Tierpsychol. 39, 178–188. doi: 10.1111/j.1439-0310.1975.tb00907.x

[ref155] Tomasello M. Farrar M. J. (1986). Joint attention and early language. Child Dev. 57, 1454–1463. doi: 10.2307/1130423, PMID: 3802971

[ref156] Townsend S. W. Manser M. B. (2013). Functionally referential communication in mammals: The past, present and the future. Ethology 119, 1–11. doi: 10.1111/eth.12015

[ref157] Vonk J. Beran M. J. (2012). Bears ‘count’ too: quantity estimation and comparison in black bears (*Ursus americanus*). Anim. Behav. 84, 231–238. doi: 10.1016/j.anbehav.2012.05.001, PMID: 22822244 PMC3398692

[ref158] Wade N. (1980). Does man alone have language? Apes reply in riddles and a horse says neigh. Science 208, 1349–1351. doi: 10.1126/science.7375943, PMID: 7375943

[ref520] Weeden J. S. Falls J. B. (1959). Differential response of male ovenbirds to recorded songs of neighboring and more distant individuals. Auk 76, 343–351.

[ref159] Weisman R. Ratcliffe L. (1987). How birds identify species information in song: a pattern recognition approach. Learn. Motiv. 18, 80–98. doi: 10.1016/0023-9690(87)90024-5

[ref160] Wheeler B. C. Fischer J. (2012). Functionally referential signals: A promising paradigm whose time has passed. Evol. Anthropol. 21, 195–205. doi: 10.1002/evan.21319, PMID: 23074065

[ref161] Wickler W. (1976). The ethological analysis of attachment. Sociometric, motivational and sociophysiological aspects. Z. Tierpsychol. 42, 12–28. PMID: 790837

[ref162] Wynn K. (1990). Children’s understanding of counting. Cognition 36, 155–193. doi: 10.1016/0010-0277(90)90003-3, PMID: 2225756

[ref400] Wynn K. (1992). Children’s acquisition of the number words and the counting system. Cogn. Psychol. 24, 220–251. doi: 10.1016/0010-0285(92)90008-P

[ref164] Yasukawa K. (1981). Song repertoires in redwinged blackbird (*Agelaius phoeniceus*): A test of the beau Geste hypothesis. Anim. Behav. 24, 114–125. doi: 10.1016/S0003-3472(81)80158-3

[ref165] Young M. E. Wasserman E. A. (2001). Entropy and variability discrimination. J. Exp. Psychol. Learn. Mem. Cogn. 27, 278–293. doi: 10.1037/0278-7393.27.1.278 11204103

